# Targeted Induction of Interferon-λ in Humanized Chimeric Mouse Liver Abrogates Hepatotropic Virus Infection

**DOI:** 10.1371/journal.pone.0059611

**Published:** 2013-03-28

**Authors:** Shin-ichiro Nakagawa, Yuichi Hirata, Takeshi Kameyama, Yuko Tokunaga, Yasumasa Nishito, Kazuko Hirabayashi, Junichi Yano, Takahiro Ochiya, Chise Tateno, Yasuhito Tanaka, Masashi Mizokami, Kyoko Tsukiyama-Kohara, Kazuaki Inoue, Makoto Yoshiba, Akinori Takaoka, Michinori Kohara

**Affiliations:** 1 Department of Microbiology and Cell Biology, Tokyo Metropolitan Institute of Medical Science, Setagaya-ku, Tokyo, Japan; 2 Discovery Research Laboratories, Nippon Shinyaku Co., Ltd., Tsukuba, Ibaraki, Japan; 3 Division of Signaling in Cancer and Immunology, Institute for Genetic Medicine, Hokkaido University, Sapporo, Hokkaido, Japan; 4 Center for Microarray Analysis, Tokyo Metropolitan Institute of Medical Science, Setagaya-ku, Tokyo, Japan; 5 Division of Molecular and Cellular Medicine, Japanese National Cancer Center Research Institute, Chuo-ku, Tokyo, Japan; 6 PhoenixBio Co., Ltd., Higashi-Hiroshima, Hiroshima, Japan; 7 Department of Virology and Liver Unit, Nagoya City University Graduate School of Medical Science, Nagoya, Aichi, Japan; 8 Research Center for Hepatitis and Immunology, International Medical Center of Japan Konodai Hospital, Ichikawa, Chiba, Japan; 9 Transboundary Animal Diseases Center, Joint Faculty of Veterinary Medicine, Kagoshima University, Kagoshima, Japan; 10 Division of Gastroenterology, Showa University Fujigaoka Hospital, Yokohama, Kanagawa, Japan; University of Tennessee Health Science Center, United States of America

## Abstract

**Background & Aims:**

The interferon (IFN) system plays a critical role in innate antiviral response. We presume that targeted induction of IFN in human liver shows robust antiviral effects on hepatitis C virus (HCV) and hepatitis B virus (HBV).

**Methods:**

This study used chimeric mice harboring humanized livers and infected with HCV or HBV. This mouse model permitted simultaneous analysis of immune responses by human and mouse hepatocytes in the same liver and exploration of the mechanism of antiviral effect against these viruses. Targeted expression of IFN was induced by treating the animals with a complex comprising a hepatotropic cationic liposome and a synthetic double-stranded RNA analog, pIC (LIC-pIC). Viral replication, IFN gene expression, IFN protein production, and IFN antiviral activity were analyzed (for type I, II and III IFNs) in the livers and sera of these humanized chimeric mice.

**Results:**

Following treatment with LIC-pIC, the humanized livers of chimeric mice exhibited increased expression (at the mRNA and protein level) of human IFN-λs, resulting in strong antiviral effect on HBV and HCV. Similar increases were not seen for human IFN-α or IFN-β in these animals. Strong induction of IFN-λs by LIC-pIC occurred only in human hepatocytes, and not in mouse hepatocytes nor in human cell lines derived from other (non-hepatic) tissues. LIC-pIC-induced IFN-λ production was mediated by the immune sensor adaptor molecules mitochondrial antiviral signaling protein (MAVS) and Toll/IL-1R domain-containing adaptor molecule-1 (TICAM-1), suggesting dual recognition of LIC-pIC by both sensor adaptor pathways.

**Conclusions:**

These findings demonstrate that the expression and function of various IFNs differ depending on the animal species and tissues under investigation. Chimeric mice harboring humanized livers demonstrate that IFN-λs play an important role in the defense against human hepatic virus infection.

## Introduction

The interferon (IFN) family of cytokines are important mediators of the innate immune response and contribute to the first line of defense against viral infection. The IFNs are classified as type I (IFN-α and IFN-β), type II (IFN-γ), or type III (IFN-λ), based on receptor complex recognition and protein structure.

Innate immune responses, such as IFN induction, contribute to defenses against microbial pathogens. Pathogens are recognized by four types of receptors associated with innate immunity: Toll-like receptors (TLRs), Nod-like receptors, retinoic acid-inducible gene-I (RIG-I) -like receptors and C-type lectins [1]. The initial recognition of pathogens by these receptors induces inflammatory reactions at the infected site, and also triggers adaptive immunity against the pathogens. Thus, activation of the innate immune response exerts antiviral effects. Immunity-associated receptor agonists are therefore good candidates for antiviral drugs and adjuvants [2].

IFN-α and IFN-β are currently employed therapeutically. The most noteworthy example is in the treatment of chronic hepatitis C virus (HCV) infection with pegylated IFN-α (PegIFN-α) [3]. These IFNs also are used against chronic hepatitis B virus (HBV) infection [4]. Some groups recently have reported that HCV infection results in expression of IFN-λs in primary human hepatocytes [5] and in the livers of chimpanzees [6]. Additionally, variation near the IFN-λ3 (IL-28B) -encoding gene is strongly associated with treatment response to pegylated IFN and ribavirin for chronic HCV infection and spontaneous eradication [7], [8], [9], [10].

To target induction of the innate immune system of liver without inducing systemic immune activation, we developed a complex of cationic liposome (LIC) and the synthetic double-stranded RNA analog (polyinosinic-polycytidylic acid; pIC), termed LIC-pIC. We have reported that LIC is a safe and effective delivery tool for oligonucleotides [11], [12]. Furthermore, we have shown, using administration in animal models, that RNA complexed with LIC can be delivered in large quantities to the liver (**Figure S1**). pIC is a well-known inducer of IFN-α and IFN-β production, a role mediated through pIC’s recognition by TLR3 [13] or RIG-I-like receptors [14].

In the present study, we demonstrate, using administration of LIC-pIC, the distinct innate immune responses of human and mouse hepatocytes in chimeric mice harboring human/mouse livers infected with HCV or HBV [15], [16]. The animals used here were transgenic severe combined immunodeficient (SCID) mice that carried additional copies of the urokinase plasminogen activator-encoding gene, resulting in the apoptosis of endogenous mouse hepatocytes, which then were replaced with human hepatocytes. This animal model provided robust HBV or HCV infection in chimeric mice harboring humanized livers. Because these rodents were T- and B-cell deficient, the mice were not appropriate for studies of adaptive immunity. Nonetheless, this model provided new insights into HCV innate host responses and therapeutic approaches. Furthermore, these models permitted analyses of human/mouse hepatocyte immune responses in the same liver. These analyses indicated that IFN-λs play a critical role in the antiviral response of human hepatocytes, and that IFN-β is induced in the response of mouse hepatocytes. Analysis of several cell lines showed that this robust IFN-λ induction was limited to the human hepatocytes. These results suggest that the function of IFN-λs, IFN-α, and IFN-β differ depending on the animal species and on the tissue under study.

## Materials and Methods

### Ethics Statement

This study was carried out in strict accordance with both the *Guidelines for Animal Experimentation* of the Japanese Association for Laboratory Animal Science and the recommendations in the *Guide for the Care and Use of Laboratory Animals* of the National Institutes of Health. All protocols were approved by the ethics committee of Tokyo Metropolitan Institute of Medical Science. The HCV-infected patient who served as the source of the serum samples provided written informed consent prior to blood collection.

### Nucleic Acids and Complexes

pIC and LIC were prepared as previously described [11]. In brief, the distribution of the chain lengths of polyinosinic and polycytidylic acids (Yamasa, Chiba, Japan) was adjusted by heating to give an apparent maximum of 200–400 bases, as determined by gel filtration high-performance liquid chromatography. Liposomes composed of the cationic lipid analogue 2-*O*-(2-diethylaminoethyl)-carbamoyl-1,3-*O*-dioleoylglycerol (synthesized at Nippon Shinyaku Co., Ltd., Kyoto, Japan) and egg phosphatidylcholine (NOF Corp., Tokyo, Japan) were lyophilized and formulated by Nippon Shinyaku Co., Ltd. A short dsRNA, a synthetic siRNA against the firefly luciferase gene, was purchased from Hokkaido System Science (Sapporo, Japan). The sequence of this dsRNA was 5′-GCUAUGAAACGAUAUGGGC-dTdT-3′ (sense) and 5′-GCCCAUAUCGUUUCAUAGC-dTdT-3′ (antisense). Atelocollagen, a highly purified type I collagen prepared by pepsin treatment of calf dermis, was obtained from Koken (Tokyo, Japan). Each nucleic acid complex with LIC or atelocollagen was freshly prepared for each experiment.

### Generation of Chimeric Mice Infected with HCV or HBV

Chimeric mice infected with HCV or HBV were prepared as previously described [17], [18]. In brief, uPA^+/+^/SCID mice transplanted with human hepatocytes were obtained from PhoenixBio (Hiroshima, Japan) [19]. Six weeks after transplantation, we injected each chimeric mouse intravenously (IV) with HCV (10^6^ copies per dose) -containing serum or HBV-containing culture supernatant. The serum was obtained from a HCV-infected patient harboring HCV genotype 1b (HCR6; accession number AY045702) or 1a (HCG9; accession number AB520610); the supernatant was concentrated from a culture containing HBV genotype C. By the time of the first drug administration, the serum HCV genomic RNA levels had reached 6.6×10^5^ to 2.7×10^7^ copies/ml (genotype 1b) or 2.9×10^6^ to 2.8×10^8^ copies/ml (genotype 1a); the serum HBV genomic DNA levels had reached 2.4×10^7^ to 1.2×10^9^ copies/ml.

### Treatment of Chimeric Mice

Virus-infected chimeric mice with humanized livers were randomly allocated to treatment groups of 3–5 mice each. Starting on Day 0, the chimeras received once- or three-times-daily IV injections (via the tail or orbital vein) of saline, LIC-pIC, atelocollagen-pIC, LIC-short dsRNA, pIC, human IFN-λ1, human IFN-λ2, or human IFN-λ3 (the human IFN-λs were from R&D Systems, Minneapolis, MN, USA) and once-weekly (Days 0 and 7) IV injections of α-galactosylceramide (KRN7000; Kirin Brewery, Gunma, Japan); twice-weekly (Days 0, 3, 7 and 10) subcutaneous (SC) injections of 30 µg/kg PegIFN-α (PegIFNα-2a; Chugai Pharmaceutical Co., Ltd, Tokyo, Japan); or once-daily peroral entecavir (ETV) (Bristol-Myers Squibb, New York, NY, USA). The IV injections were performed under gentle pressure to avoid non-specific delivery effects. All drugs were administered at a dosing volume of 5–10 µl/g of body weight.

### Quantification of HCV Genomic RNA Levels by qRT-PCR

Total RNA was purified from the serum or liver tissue of chimeric mice with humanized livers by the acid guanidinium-phenol-chloroform method. HCV genomic RNA levels were quantified by qRT-PCR with an ABI7700 sequence detector system (Applied Biosystems, Foster City, CA, USA) as previously described [20].

### Quantification of HBV Genomic DNA by qPCR

DNA extraction from the serum and liver tissue, and quantification of HBV genomic DNA, were performed as previously described [21].

### Immunofluorescence Analysis

Liver tissues obtained from mice were embedded in OCT compound. The frozen tissues were cut into thin sections (5–8 µm) and placed on glass slides. After fixation, mouse liver sections were probed with an anti-HCV core protein monoclonal antibody (5E3) labeled with biotin [22] as the primary antibody, followed by streptavidin Alexa-488 conjugate (Invitrogen Corp., Carlsbad, CA, USA). To detect human hepatocytes, liver sections were probed with anti-human hepatocyte monoclonal antibody (Dako, Glostrup, Denmark), followed by anti-mouse IgG-Alexa 546 (Molecular Probes). The nuclei were stained with 4′,6-diamidino-2-phenylindole (DAPI).

### Cells

HepG2, Huh-7, and HEK293T cells were obtained from the American Type Culture Collection. MRC-5 (RCB0218) cells were provided by the RIKEN BioResource Center through the National Bio-Resource Project of the MEXT, Japan. All cells were cultured as described [23]. Two types of human hepatoma HuH-7 cells carrying an HCV sub-genomic replicon (FLR 3-1 (genotype 1b, Con-1) and R6FLR-N (genotype 1b, strain HCR6 and N)) were used and cultured as described [24], [25].

### RNA-mediated Interference

Chemically synthesized 21-nucleotide siRNA, including control siRNA (siPerfect Negative control), was obtained from Sigma. The sequences of these RNAs are 5′-GCCAUAGACCACUCAGCUU-3′ (siTICAM-1) and 5′-CCACCUUGAUGCCUGUGAA-3′ (siMAVS) (only the sense strands are shown). Cells were transfected with 50 nM siRNA in 2.0 µl Lipofectamine RNAiMAX (Invitrogen), and then were used 48 h later for further experiments.

### Microarray Analysis

Total RNA was extracted from chimeric mouse livers using ISOGEN (Nippon Gene, Tokyo, Japan), and was purified using an RNeasy Mini Kit (QIAGEN, Valencia, CA, USA). RNA integrity was assessed with a Bioanalyzer (Agilent Technologies, Santa Clara, CA, USA). cRNA was prepared and the microarray (Agilent Technologies) was hybridized and scanned according to the manufacturer’s instructions. Whole human genome 4×44 K format microarrays (G4112F; Agilent Technologies) were used.

### Gene Expression Analysis

Total RNA or cDNA, which was synthesized from total RNA using a High Capacity cDNA Reverse Transcription Kit (Applied Biosystems), was used for qRT-PCR or qPCR performed using TaqMan Gene Expression Assays and the ABI7700 sequence detector system. The TaqMan Gene Expression Assays for *IFNB1*, *Ifnb1*, *IFNA2*, *Ifna2*, *IFNG*, *Ifng*, *IFNL1* (also known as *IL29*), *Ifnl2/3* (also known as *Il28a/b*), *IFNAR1, Ifnar1, IFNAR2, Ifnar2, IFNLR1* (also known as *IL28RA*), and *Ifnlr1* (also known as *Il28ra*) were obtained from Applied Biosystems. The primers and probes for the *IFNL2* and *IFNL3* (also known as *IL28A* and *IL28B*, respectively) genes were obtained from Takara Bio, Inc. (Shiga, Japan). The primers and probes for the *IFNL2* gene consisted of a forward primer, IL28A-207-S21 (nt 207–227; 5′-GCTGAAGGACTGCAGGTGCCA-3′); a reverse primer, IL28A-378-R20 (nt 359–378; 5′-GGGCTGGTCCAAGACGTCCA-3′); and a TaqMan probe, IL28A-286-S24FT (nt 286–309; 5′-ATGGCTTTGGAGGCTGAGCTGGCC-3′). The primers and probes for the *IFNL3* gene consisted of a forward primer, IL28B-207-S21 (nt 207–227; 5′-GCTGAAGGACTGCAAGTGCCG-3′); a reverse primer, IL28B-379-R21 (nt 359–379; 5′-GGGGCTGGTCCAAGACATCCC-3′); and a TaqMan probe, IL28B-286-R20FT (nt 267–286; 5′-CGGGGCGCTCCCTCACCTGC-3′). A reporter dye, 6-carboxy-fluorescein, was covalently attached to the 5′-end of the probe sequence and a quencher dye, 6-carboxytetramethylrhodamine, was attached to its 3′-end.

Calibration curves for quantification of the IFN-α- or IFN-β-encoding genes and the IFN-λs-encoding genes were prepared by using a series of ten-fold dilutions of human *IFNB1* and *IFNA2* RNAs (synthesized in our laboratory), and human *IFNL1*, *IFNL2*, and *IFNL3* cDNA clones (Open Biosystems, Inc., Huntsville, AL, USA). Each assay specifically detected its own target.

Quantification of *IFNAR1/2, Ifnar1/2, IFNLR1,* and *Ifnlr1* mRNA was estimated using the calibration curves for the *IFNL1* cDNA.

### IFN-λ Neutralization Studies

IFN-λ1 to IFN-λ3 in the chimeric mice were neutralized by daily IV injection of 3 mg/kg anti-human IFN-λ1 (R&D Systems) and 1 mg/kg anti-human IFN-λ2 (R&D Systems) antibodies.

### Statistical Analysis

The data were analyzed with SAS System version 8.2 (SAS Institute, Inc., Cary, NC, USA). *P* values of ≤0.05 were considered significant.

## Results

### Antiviral Responses Elicited by LIC-pIC in HCV- or HBV-infected Chimeric Mice with Humanized Livers

To confirm that HCV and HBV can infect humanized chimeric mice, we measured HCV RNA or HBV DNA levels in serum weekly after inoculation with the respective virus (**Figure S2A and S2B**). At 3 weeks post-infection, HCV-RNA levels reached 10^6^–10^7^ copies/mL in the genotype 1b group (**Figure S2A**); at 9 weeks post-infection, HBV-DNA levels reached 10^7^–10^8^ copies/mL (**Figure S2B**). These results demonstrated that HCV or HBV can replicate in, and persistently infect, human hepatocytes transplanted into chimeric mice.

**Figure 1:**
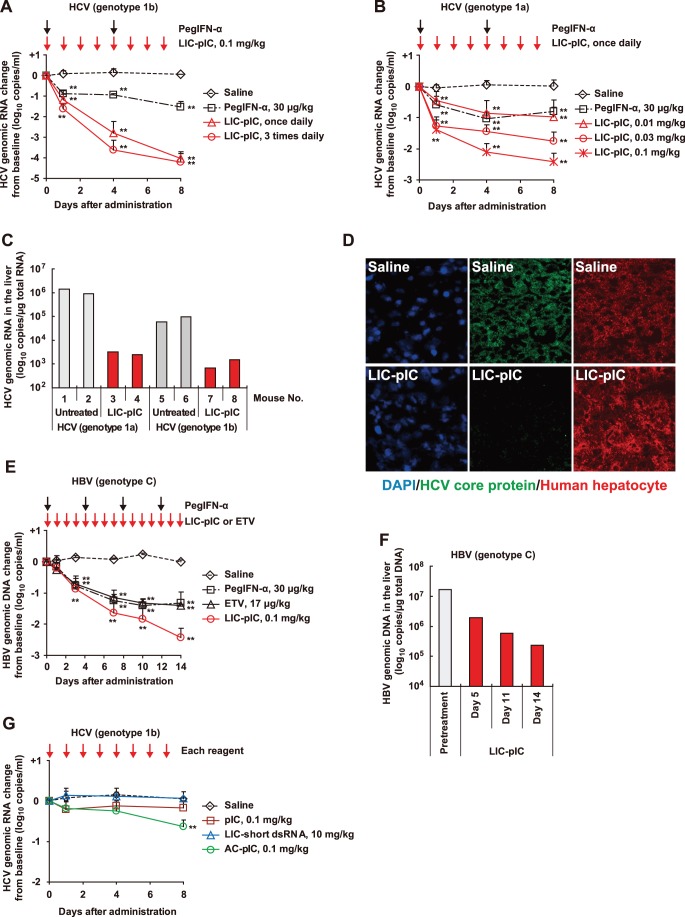
Antiviral responses elicited by LIC-pIC in chimeric mice with humanized livers. (**A**, **B**) Serum HCV RNA levels, relative to the baseline, in HCV genotype 1b-infected (**A**) or 1a-infected (**B**) chimeric mice following the indicated treatments, as determined by qRT-PCR (*n* = 3–5 per group). (**C**) Liver HCV RNA levels on Day 4 of LIC-pIC treatment (0.1 mg/kg/day), as determined by qRT-PCR. (**D**) Immunostained liver tissues of HCV genotype 1b-infected chimeric mice treated with saline (Upper) and LIC-pIC (0.1 mg/kg/day) (Lower) at Day 4. (**E**) Serum HBV DNA levels relative to the baseline following the indicated treatments, as determined by qPCR (*n* = 3–4 per group). (**F**) HBV DNA levels of chimeric mouse livers following LIC-pIC treatment, as determined by qPCR (*n* = 1 per each time point, the results indicates the mean of two measurements) (**G**) HCV RNA levels relative to the baseline following the indicated treatments, as determined by qRT-PCR (*n* = 3–4 per group). In all cases, bars indicate SD. **P<0.01, treatment vs. saline control by Dunnett’s multiple comparison test.

Treatment of HCV genotype 1b-infected chimeric mice with LIC-pIC (0.1 mg/kg; 1 or 3 times/day) led to a dose-dependent reduction in serum HCV RNA that was greater than the reduction induced by PegIFN-α treatment (30 µg/kg; twice/week, 20-fold the typical clinical dose) (**Figure 1A**). Treatment of HCV genotype 1a-infected mice with LIC-pIC (0.01, 0.03 or 0.1 mg/kg; once/day) had a similar antiviral effect (**Figure 1B**). Treatment of these mice with LIC-pIC also reduced liver HCV RNA levels (**Figure 1C**) and liver HCV core protein levels (**Figure 1D**).

Treatment with LIC-pIC (0.1 mg/kg/day) also reduced serum HBV DNA levels in HBV-infected mice more effectively than entecavir (ETV) treatment (17 µg/kg/day; the same as the clinical dose) by Day 14 (**Figure 1E**) and additionally reduced liver HBV DNA levels (**Figure 1F**). LIC-pIC complex treatment therefore elicited stronger antiviral responses against both HCV and HBV than currently used antiviral agents without marked hepatotoxicity (**Figure S3**).

We next analyzed the effect on serum HCV RNA levels of treatment with pIC alone; with pIC complexed with the non-hepatotropic carrier atelocollagen (AC) [26]; or with a short dsRNA complexed with LIC. Significant reductions in serum HCV RNA levels were observed only following treatment with the AC-pIC complex (**Figure 1G**), indicating that the active ingredient of LIC-pIC was pIC and that the antiviral effects of pIC were enhanced by complexing with LIC.

### Expression of IFN-α, IFN-β and IFN-γ Induced by LIC-pIC in Chimeric Mice with Humanized Livers

**Figure 2:**
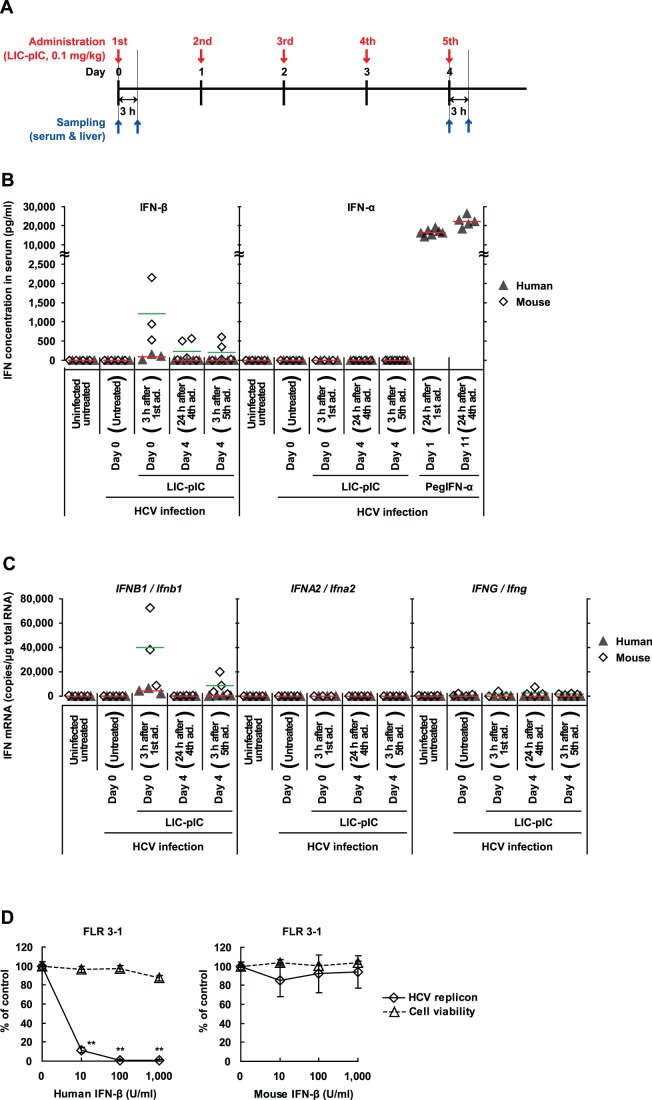
Expression of genes encoding IFN-α, IFN-β, and IFN-γ following induction by LIC-pIC in chimeric mice with humanized livers. (**A**) Experimental schedule in chimeric mice infected with HCV. (**B**) Serum concentrations (in chimeric mice) of human and mouse IFN-β and IFN-α, as determined by ELISA (*n* = 3–5 for no treatment or LIC-pIC group; *n* = 6–8 for PegIFN-α group). Bars indicate the mean concentration of human (red) and mouse (green) IFNs averaged across the entire group. The results indicates the representative of two measurements (**C**) The liver mRNA levels of genes encoding IFN-β, IFN-α2 and IFN-γ (human genes: *IFNB1*, *IFNA2* and *IFNG*, respectively; mouse genes: *Ifnb1*, *Ifna2* and *Ifng*, respectively), as determined by qRT-PCR (*n* = 3–4 per group). The bars indicate the mean mRNA levels of human (red) and mouse (green) IFN genes averaged across the entire group. The results indicates the representative of two measurements (**D**) Effectiveness of recombinant human or mouse IFN-β against HCV in FLR 3-1 HCV-replicon cells. The bars indicate SD (*n* = 4 per group). **P<0.01, treatments vs. the vehicle by Dunnett’s multiple comparison test.

pIC induces IFN-α and IFN-β production through recognition by TLR3 [13] or RIG-I-like receptors [14]. To examine whether the potent antiviral responses elicited by LIC-pIC were caused by its reported ability to induce IFN-α and IFN-β, we analyzed the expression levels of these IFNs after administration of 0.1 mg/kg LIC-pIC to virally infected mice. Administration and sampling were conducted according to the schedules shown in **Figure 2A** (HCV-infected mice) and **Figure S4A (**HBV-infected mice). Because the mouse livers used in this study were human-mouse chimeras (average substitution rate ≈80%) [19], we measured the induction levels of both human and mouse IFNs.

The first administration of LIC-pIC (Day 0) induced both human and mouse IFN-β in the serum, as well as *IFNB1* (human) and *Ifnb1* (mouse) mRNA in the liver (**Figure 2B and 2C**). The mRNA induction levels peaked 3 h after the first administration and decreased to the pretreatment levels within 24 h (data not shown). On repeated administration, the peak levels of both protein and mRNA fell gradually in the mouse, whereas induction of humanized liver-derived IFN products was observed only at the first administration (**Figure 2B and 2C**), although the antiviral responses elicited by LIC-pIC were sustained throughout the administration period. In addition, we did not observe induction of human or mouse IFN-α in the serum, or of *IFNA2* or *IFNG* (human) or *Ifna2* or *Ifng* (mouse) mRNA in the liver, at any point during LIC-pIC administration (**Figure 2B and 2C**, and **Figure S4B** and S**4C**). The induced mouse IFN-β that was still observed after the fifth administration was not expected to have any antiviral effect because mouse IFN-β does not suppress HCV replication in HCV replicon-containing cells (**Figure 2D**), indicating that mouse IFN-β does not activate signaling by the human receptor. Thus, the kinetics of IFN-α, IFN-β, and IFN-γ induction by LIC-pIC were inconsistent with the duration of the antiviral responses that were observed.

Moreover, the maximum serum concentration of human IFN-β that was induced by LIC-pIC was only 92 pg/ml (**Figure 2B**), which was 13-fold lower than the serum level of mouse IFN-β, despite the high human hepatocyte substitution rate in the liver. This poor induction of human IFN-β by LIC-pIC was independent of HCV infection (**Figure S5**). As for IFN-α, no serum IFN-α could be detected following LIC-pIC treatment, whereas a constant high concentration of human IFN-α (≈20,000 pg/ml) was observed in the serum of chimeric mice treated with PegIFN-α (**Figure 2B**). Thus, the concentration of IFN-α and IFN-β in the serum of chimeric mice treated with LIC-pIC could not explain the potency of the LIC-pIC-induced antiviral response.

Although pIC is also known as an activator of natural killer (NK) or NKT cells [27], [28], these cell types were not involved in the antiviral response elicited by LIC-pIC in the virus-infected chimeric mice of this study (**Figure S6**). These cumulative data indicated that an unknown mechanism was responsible for the observed antiviral response.

### IFN-λs Mediate the Antiviral Response of Chimeric Mice with Humanized Livers to LIC-pIC

**Figure 3:**
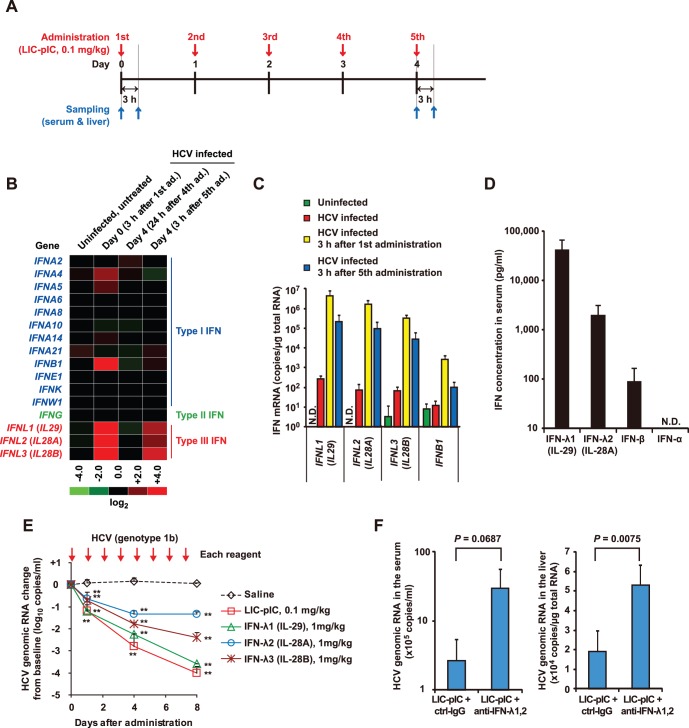
IFN expression following induction of innate immunity by LIC-pIC. (**A**) Experimental schedule in the chimeric mice infected with HCV. (**B**) Microarray analysis of IFN family gene expression. Log_2_ ratio values of gene expression were calculated with respect to control (untreated and HCV-infected) chimeric mice**.** (**C**) Liver mRNA levels of human IFN genes (*n* = 3–5 per group), as determined by qRT-PCR. (**D**) Serum concentration of human IFN-λ1, IFN-λ2, IFN-β, and IFN-α (*n* = 3 per group), as determined by ELISA. (**E**) Serum HCV RNA levels, relative to the baseline, in HCV-infected chimeric mice treated with human IFN-λ, LIC-pIC, or control saline (*n* = 3–5 per group), as determined by qRT-PCR. (**F**) The HCV RNA levels in the serum and liver of HCV genotype 1a-infected chimeric mice collected on Day 4 of LIC-pIC treatment, as determined by qRT-PCR. The mice were additionally treated with either a control antibody or a neutralizing antibody against IFN-λ (*n* = 3 per group). Bars indicate SD. **P<0.01, treatments vs. saline controls on the respective day, by Dunnett’s multiple comparison test.

To determine the mechanism of the LIC-pIC-induced antiviral response, we performed comprehensive gene expression analysis to identify genes whose expression kinetics were consistent with the duration of this response. DNA microarray analysis of the livers of chimeric mice infected with HCV and treated, or not, with LIC-pIC identified type III IFN genes as having suitable expression kinetics (**Figure 3A and 3B**). There are three type III IFN genes in humans, *IFNL1*, *IFNL2*, and *IFNL3* (formerly *IL29*, *IL28A,* and *IL28B*, respectively), which encode IFN-λ1, IFN-λ2 and IFN-λ3 (also designated IL-29, IL-28A, and IL-28B), respectively [29], [30]. The genes that were analyzed included those encoding IFN-α, IFN-β, IFN-ε, IFN-κ, IFN-ω, IFN-γ, and IFN-λ. Among these genes, the three IFN-λ-encoding genes were the only ones whose levels remained up-regulated 3 h after the fifth LIC-pIC administration to HCV-infected mice on Day 4 (**Figure 3B**). qRT-PCR demonstrated that, unlike *IFNB1*, the mRNA levels of human *IFNL1* (*IL29*), *IFNL2* (*IL28A*), and *IFNL3* (*IL28B*) were strongly induced in the livers of chimeric mice treated with LIC-pIC (**Figure 3C**). In addition, expression of human *IFNL1*, *IFNL2*, and *IFNL3* was induced at each LIC-pIC administration. Consistent with these gene expression results, ELISA analysis revealed that the serum levels of IFN-λ1 and IFN-λ2 were much higher than the level of IFN-β (**Figure 3D**). These results indicated that the expression of IFN-λs was strongly and persistently induced by LIC-pIC in human hepatocytes transplanted into chimeric mice.

We further investigated whether IFN-λs directly affected HCV or HBV replication. Indeed, recombinant human IFN-λ1, IFN-λ2, and IFN-λ3, when added to HCV replicon cells, suppressed virus replication in a concentration-dependent manner (**Figure S7**). Furthermore, chimeric mice dosed with IFN-λs had (by Day 8) serum HCV RNA levels that were significantly lower than those of the saline-treated control group (**Figure 3E**). Treatment with IFN-λ2 also reduced serum HBV DNA levels compared to those seen in the saline-treated control group by Day 7 (**Figure S8**). Furthermore, combination treatments with neutralizing antibodies against IFN-λ1 and IFN-λ2 (a treatment that neutralizes all three IFN-λ subtypes due to cross-reactivity (**Figure S9**)) attenuated the LIC-pIC-dependent reduction in HCV RNA levels in both serum and liver (**Figure 3F**). These results demonstrated that IFN-λs play a critical role in the antiviral response elicited in human hepatocytes by LIC-pIC.

### The Induction of IFN-λs by LIC-pIC and Antiviral Effect in Humanized Chimeric Mice with Genetic Variants of IFNL3 (IL28B)

Patients with the G-allele of rs8099917, a SNP that is located near the *IFNL3* gene, are less likely to spontaneously clear acute HCV infection and respond to combination therapy with PegIFN-α and ribavirin [7], [8], [9], [10]. We examined whether this genetic variation influenced the induction of IFN-λs in human hepatocytes and antiviral response to HCV. In the present study, LIC-pIC induced IFN-λ gene expression and anti-HCV effects to the same degree, even in chimeric mice harboring human hepatocytes derived from donors bearing the rs8099917 G-allele (**Figure S10A** and **10B**). These results indicate that neither induction of IFN-λs by LIC-pIC nor antiviral response was significantly influenced by this genomic polymorphism.

### The Difference in Innate Immunity Induced by LIC-pIC in Human and Mouse Livers

The present study used chimeric mice with humanized livers, a model that permitted simultaneous analysis of human and mouse gene expression in the same liver. With LIC-pIC treatment, expression of IFN-λ-encoding genes was induced to levels greater than those of IFN-α- and IFN-β-encoding genes in human hepatocytes, whereas *Infb1* was induced to levels greater than expressions of IFN-λ-encoding genes in mouse hepatocytes (**Figure S11A**). Moreover, gene expression analysis indicated that the expression patterns of the genes encoding the corresponding IFN receptors differed between the human and mouse hepatocytes. Specifically, the genes encoding the IFN-α and IFN-β receptors were more strongly expressed in mouse hepatocytes (as *Ifnar1* and *Ifnar2*, respectively) than in human hepatocytes (as *IFNAR1* and *IFNAR2*, respectively), whereas the gene encoding the IFN-λ receptor was more strongly expressed in human hepatocytes (as *IFNLR1* (formerly *IL28RA*)) than in mouse hepatocytes (as *Ifnlr1* (formerly *Il28ra*)) (**Figure S11B**, **S11C**, and **S11D**). These results strongly suggest that IFN-λs play a critical role in the immune response by human hepatocytes, while IFN-α and IFN-β are important for the response by mouse hepatocytes.

### IFN Expression in Human Cell Lines Following the Induction of Innate Immunity with Various Stimuli

**Figure 4:**
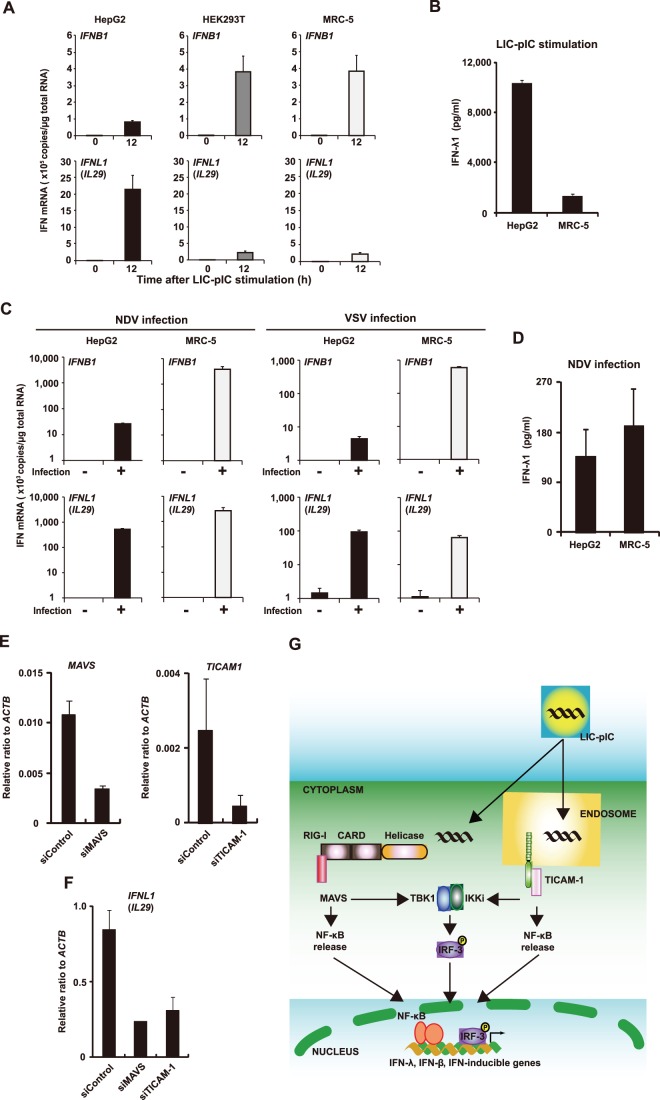
IFN expression in cell lines following the induction of innate immunity by various stimuli. (**A**, **B**) The indicated cell lines were stimulated by exposure to LIC-pIC (1 µg/ml). (**A**) The mRNA levels of IFN-β- and IFN-λ1-encoding genes (*IFNB1* and *IFNL1* (*IL29*), respectively) before and after (12 h) stimulation, as determined by qRT-PCR. (**B**) The concentration of IFN-λ1 (IL-29) in the culture medium 24 h after stimulation, as determined by ELISA. (**C**, **D**) The indicated cell lines were stimulated by viral infection with NDV or VSV. (**C**) The mRNA levels of IFN-β- and IFN-λ1-encoding genes (*IFNB1* and *IFNL1* (*IL29*), respectively) in HepG2 cells and MRC-5 cells in the absence or presence (12 h) of NDV or VSV, as determined by qRT-PCR. (**D**) The concentration of IFN-λ1 protein in the culture medium at 24 h post-infection, as determined by ELISA. (**E**, **F**) Expression in HepG2 cells of genes encoding MAVS, TICAM-1, and IFN-λ1 (*MAVS, TICAM1,* and *IFNL1* (*IL29*) respectively), as determined by qRT-PCR, after 12 h stimulation with LIC-pIC (1 µg/ml). Cells were pre-treated for 48 h with siRNAs against *MAVS*, *TICAM-1*, or a non-target control. Bars indicate SD (*n* = 3 per group). (**G**) Schematic outline of the proposed mechanism of the antiviral response induced by LIC-pIC.

To provide a comparison with the innate immune response of human hepatocytes, we investigated the role of IFNs in cell lines derived from other tissues. For this purpose, we examined the expression levels of the genes encoding IFN-λ and IFN-β following treatment with LIC-pIC in several human cell lines. In response to treatment with LIC-pIC, *IFNL1* mRNA was more strongly induced in HepG2 cells, a hepatocyte cell line, than in HEK293T or MRC-5 cells, kidney and fibroblast cell lines, respectively; the opposite expression pattern was observed for the induction of *IFNB1* mRNA (**Figure 4A**). Indeed, the protein level of IFN-λ1 in cell culture medium was also much higher in HepG2 cells than in MRC-5 cells (**Figure 4B**). Interestingly, whereas LIC-pIC induction of *IFNL1* mRNA and IFN-λ1 protein was weaker in MRC-5 cells than in HepG2 cells, the levels of *IFNL1* mRNA and IFN-λ1 protein in MRC-5 cells were almost comparable to those seen in HepG2 cells upon infection with Newcastle disease virus (NDV) or vesicular stomatitis virus (VSV) (**Figure 4C and 4D**). These data suggest that the *IFNL1*/IFN-λ1 expression profile induced by LIC-pIC was different from that induced upon viral infection. Using siRNA technology, we found that the expression of *IFNL1* induced by LIC-pIC was mediated by both MAVS (the adaptor molecule that links RIG-I-like receptor signaling pathways) and TICAM-1 (a TLR adaptor molecule) (**Figure 4E and 4F**). These results suggest that the innate immune response of human hepatocytes has unique characteristics in terms of IFN responses, and that LIC-pIC can induce robust production of IFN-λs by human hepatocytes through recognition by two distinct innate immune molecules (**Figure 4G**).

## Discussion

In this study, we demonstrated that treatment of chimeric mice (harboring human hepatocytes) with LIC-pIC resulted in the efficient induction of innate immunity that was associated with robust production of IFN-λs and resulted in the abrogation of infection by hepatotropic viruses such as HCV and HBV. Recently, two different groups, Marukian *et* *al*. [5] and Thomas *et* *al*. [6], have reported that HCV infection results in production of IFN-λs in primary human hepatocytes and in the livers of chimpanzees, providing control of HCV replication. In the present study, we extend those analyses by showing that the activation of the intrinsic immune system in human hepatocytes induces the robust production of IFN-λs, thereby providing efficient defense against viral pathogens. Furthermore, we show that neither induction of IFN-λs by LIC-pIC nor associated antiviral effects are influenced significantly by a genomic polymorphism near the *IFNL3* gene. In contrast, the efficacy of combination therapy of PegIFN-α and ribavirin varies with genomic variations at this site. More recently, Prokunina-Olsson *et al*. have demonstrated that a dinucleotide variant, ss469415590 (TT or ΔG), located upstream of *IFNL3* is strongly associated with HCV clearance, and that its frameshift variant ss469415590[ΔG] creates a new gene, termed *IFNL4* [31]. These findings may provide a new approach to investigate whether treatment with LIC-pIC induces *IFNL4* and whether the antiviral effects of LIC-pIC are influenced by the induction of *IFNL4*.

To date, several mouse studies have indicated that IFN-λs play an important role in host defense against pathogens in vaginal, intestinal, and tracheal epithelial cells [32], [33], [34], but that these IFN molecules do not contribute to innate immunity against hepatotropic viruses [35]. In the present study, we showed that LIC-pIC treatment activated the innate immune response of human hepatocytes in chimeric mouse liver, providing robust expression of the IFN-λ-encoding gene without significantly inducing expression of the IFN-β-encoding gene. Moreover, we showed that human hepatocytes expressed the IFN-λ receptor-encoding genes at high levels, while expression of the IFN-β receptor-encoding genes remained low. In contrast, expression of the IFN-λ receptor-encoding genes was low and expression of the IFN-β receptor-encoding genes was high in mouse hepatocytes from the same livers. Thus, human hepatocytes (in contrast to mouse hepatocytes) are rich sources of IFN-λs, while IFN-α or IFN-β are produced at lower levels in these cells. Indeed, the expression (in humanized livers) of these IFN-λ-encoding genes remained upregulated through at least five daily LIC-pIC administrations to chimeric mice infected with HCV. Consistent with this bias in IFN production, human hepatocytes were expected to be more receptive to stimulation by IFN-λs than their mouse counterparts.

We demonstrated the robust induction (by LIC-pIC) of IFN-λ rather than IFN-α or IFN-β in human hepatocytes. In several human cell lines derived from other tissues, IFN-λ1 induction by LIC-pIC stimulation was reduced in comparison to that seen in human hepatocytes, indicating that induction of IFN-based innate immunity differs among tissues and that human hepatocytes are high producers of IFN-λs. We also demonstrated that NDV and VSV, ssRNA viruses that are recognized by cytoplasmic sensors (RIG-I-like receptors) [36], could induce the expression of IFN-λs in several human cell lines. However, in contrast to LIC-pIC stimulation, the expression level of IFN-λ1 induced by these viruses was as low in HepG2 hepatocyte cells as in MRC-5 fibroblast cells. These results motivated us to investigate which sensors recognize LIC-pIC resulting in robust IFN-λ production. The presence of two distinct viral sensing innate immune pathways (TLR3 and RIG-I-like receptors ) in human hepatocytes has been reported [37], [38]. We showed here that the effects of LIC-pIC were mediated by both MAVS, a signaling adaptor molecule of RIG-I-like receptors, and TICAM-1, a TLR adaptor molecule. Given that both NDV and VSV effects are mediated by MAVS, we speculate that the strong induction of IFN-λs by LIC-pIC reflects dual recognition of LIC-pIC by RIG-I-like receptors and TLR3. In this context, we note that both MAVS and TICAM-1 are direct targets for proteolytic degradation by the HCV-encoded NS3/4A protease [39], [40]. Related reports [39], [40], [41] suggest that the inhibitory effect of this viral protease may affect the induction of IFN-λs, particularly in HCV-infected hepatocytes. However, we observed significantly higher levels of IFN-λ expression in response to treatment with LIC-pIC, regardless of *in vivo* HCV infection status (Fig. 3 and data not shown), suggesting that the *in vivo* induction of IFN- λs by LIC-pIC treatment may not be affected by this viral protease in our experimental system. Further analyses will be needed to clarify the balance between viral evasion and activation of the innate immune response.

Our results demonstrate that the dominant IFN systems exhibit tissue- and animal-specific induction patterns, and that IFN-λs plays a critical role in innate immune response in human hepatocytes. These findings expand our understanding of the role of IFN-λs in the human immune system.

## Supporting Information

Figure S1 **Hepatotropic property of LIC.** Biodistribution of RNA following administration of a complex of LIC and ^3^H-labeled short dsRNA to normal mice.
(EPS)

Figure S2 **Time course studies in 3 mice inoculated with HCV genotype 1b or HBV genotype C.** (**A**) HCV RNA levels in chimeric mouse serum after inoculation. (**B**) HBV DNA in chimeric mouse serum after inoculation.
(EPS)

Figure S3 **Hepatotoxicity of the LIC-pIC complex.** (**A**, **B**) Chimeric mice infected with HCV genotype 1a were treated daily for 8 days (Days 0–7) with LIC-pIC at 0.01, 0.03, or 0.1 mg/kg. The sera of chimeric mice were collected on the indicated days following the initial administration of LIC-pIC and the concentration of human albumin (**A**) or alanine transferase (ALT) (**B**) was determined (*n* = 3–5 per group).
(EPS)

Figure S4 **Expression of IFN-α, IFN-β, and IFN-γ in HBV-infected chimeric mice with humanized livers following treatment with LIC-pIC.** (**A**) Schedule of the LIC-pIC treatments and serum or liver sampling in the HBV-infected chimeric mice. (**B**) Serum concentrations of human and mouse IFN-β and IFN-α, as determined by ELISA. (**C**) The liver mRNA levels of genes encoding IFN-β, IFN-α2 and IFN-γ (human genes: *IFNB1*, *IFNA2* and *IFNG*, respectively; mouse genes: *Ifnb1*, *Ifna2* and *Ifng*, respectively), as determined by qRT-PCR. For (**B**) and (**C**), each point indicates the value for a single chimeric mouse; groups consisted of 1–4 mice each.
(EPS)

Figure S5 **Expression of genes encoding human IFN-β (** ***IFNB1*** **) and mouse IFN-β (** ***Ifnb1*** **) in HCV-infected or uninfected chimeric mice with humanized livers following treatment with LIC-pIC, as determined by qRT-PCR.** The data points indicate the mRNA levels in individual chimera; the horizontal bars indicate the mean transcript concentrations for the human (red) and mouse (green) genes (*n* = 3 per group).
(EPS)

Figure S6 **Mouse NK and NKT cells are not involved in the anti-HCV effects of LIC-pIC.** Chimeric mice with humanized livers were infected with HCV genotype 1b and treated for 14 days (Days 0–13) with one of the following: saline, daily; 1 µg/kg α-galactosylceramide (αGalCer, a specific activator of NKT cells), weekly (Days 0 and 7); or 0.1 mg/kg LIC-pIC, daily. Half of the chimeras receiving the LIC-pIC treatment were pre-treated with a TM-β1 antibody in order to deplete NK and NKT cells. The other half were left untreated (ctrl). The serum HCV genomic RNA levels were determined using qRT-PCR on the indicated days after the initial treatment was administered. Data points indicate mean values and the bars in­­­dicate SD (*n* = 4–5 per group). *P<0.05 and **P<0.01, treatments vs. the saline control by Dunnett’s multiple comparison test.
(EPS)

Figure S7 **The anti-HCV response elicited by IFN-λ in HCV replicon cells.** FLR3-1 HCV replicon cells were treated with the indicated recombinant human IFN-λs or with saline control for 72 h, and HCV replication (diamonds) and cell viability (triangles) of the cells then were assessed. Values are expressed as a percentage of the untreated control (*n* = 4 per group). Data are mean values and the bars indicate SD. **P<0.01, IFN treatments vs. the vehicle by Dunnett’s multiple comparison test.
(EPS)

Figure S8 **The anti-HBV response elicited by IFN-λ.** Serum HBV genomic DNA levels were assayed in chimeric mice infected with HBV and administered daily treatments of recombinant human IFN-λ2 or LIC-pIC. HBV levels were assayed on the indicated days after the initial treatments. Values are expressed relative to the baseline value. Data points indicate mean values and the bars indicate SD (*n* = 3–5 per group). **P<0.01, treatments vs. the saline control by Dunnett’s multiple comparison test.
(EPS)

Figure S9 **Neutralizing activity of anti-human IFN-λ antibodies.** FLR 3-1 HCV replicon cells were pre-treated with 1 µg/ml or 10 µg/ml of anti-human IFN-λ1 or anti-human IFN-λ2 antibodies before the addition of recombinant human IFN-λ1, IFN-λ2, or IFN-λ3 to the culture medium. The replication of HCV replicons was determined by luciferase assays (red bars), and cell viability was determined using a WST-8 assay (gray bars). Values are expressed as a percentage of the untreated control. Data points are mean values and the error bars indicate SD (*n* = 4 per group).
(EPS)

Figure S10 **Induction of IFN-λs by LIC-pIC and antiviral effect in human hepatocytes with genetic variants near the** ***IFNL3*** **(** ***IL28B)*** **gene.** Chimeric mice were transplanted with human hepatocytes from donors bearing a T- or G-allele of the rs8099917 (*IFNL3*-proximal) SNP. Animals were infected with HCV genotype 1b and treated daily with LIC-pIC. (**A**) The liver mRNA levels of human IFN-λ-encoding genes (*IFNL1*, *IFNL2*, and *IFNL3* (also known as *IL29, IL28A,* and *IL28B*, respectively)) were assayed by qRT-PCR. (**B**) The serum HCV genomic RNA levels were assayed by qRT-PCR. Values are expressed relative to the baseline value. Data points are presented as mean values with SD (*n* = 1–6 per group). **P<0.01, treatments vs. the saline control by Dunnett’s multiple comparison test.
(EPS)

Figure S11 **The expression of mouse IFN genes and corresponding receptors in chimeric mice with humanized livers.** (**A**) The liver mRNA levels of mouse IFN-encoding genes (*Ifnl2* (*Il28a*), *Ifnl3* (*Il28b*), and *Ifnb1*) (*n* = 3–5 per group). (**B**, **C**, **D**) The liver mRNA levels of human and mouse IFN receptor-encoding genes (mouse/human genes are *IFNAR1*/*Ifnar1, IFNAR2/Ifnar2*, and *IFNLR1*/*Ifnlr1* (*IL28RA*/*Il28ra*), respectively) (*n* = 8–9 per group) in uninfected chimeric mice (HCV-) and in chimeric mice infected with HCV genotype 1a (HCV+). Bars indicate SD. *P*-values were determined using unpaired *t*-tests.
(EPS)

Text S1 **Supporting materials, methods, and references.** Methods for “Biodistribution of nucleic acids complexed with LIC”, “Measurement of human serum albumin”, “Measurement of alanine aminotransferase”, “Enzyme-linked immunosorbent assay”, “Luciferase and WST-8 assays of HCV replicon cells”, “Depletion of NK and natural killer T cells”, “Activation of NKT cells”, “Neutralizing activity of the anti-human IFN-λ antibody”, and “SNP genotyping of *IFNL3* (*IL28B*)”, along with supporting references, are provided in the Supporting Materials section.
(DOC)

## References

[1] Kumar H, Kawai T, Akira S (2011) Pathogen recognition by the innate immune system. Int Rev Immunol 30: 16–34. 21235323 10.3109/08830185.2010.529976

[2] Akira S (2011) Innate immunity and adjuvants. Philos Trans R Soc Lond B Biol Sci 366: 2748–2755. 21893536 10.1098/rstb.2011.0106 PMC3146784

[3] Fried MW, Shiffman ML, Reddy KR, Smith C, Marinos G, et al (2002) Peginterferon alfa-2a plus ribavirin for chronic hepatitis C virus infection. N Engl J Med 347: 975–982. 12324553 10.1056/NEJMoa020047

[4] Yuen MF, Hui CK, Cheng CC, Wu CH, Lai YP, et al (2001) Long-term follow-up of interferon alfa treatment in Chinese patients with chronic hepatitis B infection: The effect on hepatitis B e antigen seroconversion and the development of cirrhosis-related complications. Hepatology 34: 139–145. 11431745 10.1053/jhep.2001.25273

[5] Marukian S, Andrus L, Sheahan TP, Jones CT, Charles ED, et al (2011) Hepatitis C virus induces interferon-lambda and interferon-stimulated genes in primary liver cultures. Hepatology 54: 1913–1923. 21800339 10.1002/hep.24580 PMC3219820

[6] Thomas E, Gonzalez VD, Li Q, Modi AA, Chen W, et al (2012) HCV Infection Induces a Unique Hepatic Innate Immune Response Associated with Robust Production of Type III Interferons. Gastroenterology 142: 978–988. 22248663 10.1053/j.gastro.2011.12.055 PMC3435150

[7] Ge D, Fellay J, Thompson AJ, Simon JS, Shianna KV, et al (2009) Genetic variation in IL28B predicts hepatitis C treatment-induced viral clearance. Nature 461: 399–401. 19684573 10.1038/nature08309

[8] Rauch A, Kutalik Z, Descombes P, Cai T, Di Iulio J, et al.. (2010) Genetic variation in IL28B is associated with chronic hepatitis C and treatment failure: a genome-wide association study. Gastroenterology 138: 1338–1345, 1345 e1331–1337. 10.1053/j.gastro.2009.12.056 20060832

[9] Suppiah V, Moldovan M, Ahlenstiel G, Berg T, Weltman M, et al (2009) IL28B is associated with response to chronic hepatitis C interferon-alpha and ribavirin therapy. Nat Genet 41: 1100–1104. 19749758 10.1038/ng.447

[10] Tanaka Y, Nishida N, Sugiyama M, Kurosaki M, Matsuura K, et al (2009) Genome-wide association of IL28B with response to pegylated interferon-alpha and ribavirin therapy for chronic hepatitis C. Nat Genet. 41: 1105–1109. 10.1038/ng.449 19749757

[11] Hirabayashi K, Yano J, Inoue T, Yamaguchi T, Tanigawara K, et al (1999) Inhibition of cancer cell growth by polyinosinic-polycytidylic acid/cationic liposome complex: a new biological activity. Cancer Res 59: 4325–4333. 10485480

[12] Yano J, Hirabayashi K, Nakagawa S, Yamaguchi T, Nogawa M, et al (2004) Antitumor activity of small interfering RNA/cationic liposome complex in mouse models of cancer. Clin Cancer Res 10: 7721–7726. 15570006 10.1158/1078-0432.CCR-04-1049

[13] Alexopoulou L, Holt AC, Medzhitov R, Flavell RA (2001) Recognition of double-stranded RNA and activation of NF-kappaB by Toll-like receptor 3. Nature 413: 732–738. 11607032 10.1038/35099560

[14] Yoneyama M, Fujita T (2009) RNA recognition and signal transduction by RIG-I-like receptors. Immunol Rev 227: 54–65. 19120475 10.1111/j.1600-065X.2008.00727.x

[15] Dandri M, Burda MR, Torok E, Pollok JM, Iwanska A, et al (2001) Repopulation of mouse liver with human hepatocytes and in vivo infection with hepatitis B virus. Hepatology 33: 981–988. 11283864 10.1053/jhep.2001.23314

[16] Mercer DF, Schiller DE, Elliott JF, Douglas DN, Hao C, et al (2001) Hepatitis C virus replication in mice with chimeric human livers. Nat Med 7: 927–933. 11479625 10.1038/90968

[17] Nakagawa S, Umehara T, Matsuda C, Kuge S, Sudoh M, et al (2007) Hsp90 inhibitors suppress HCV replication in replicon cells and humanized liver mice. Biochem Biophys Res Commun 353: 882–888. 17196931 10.1016/j.bbrc.2006.12.117

[18] Sugiyama M, Tanaka Y, Kato T, Orito E, Ito K, et al (2006) Influence of hepatitis B virus genotypes on the intra- and extracellular expression of viral DNA and antigens. Hepatology 44: 915–924. 17006908 10.1002/hep.21345

[19] Tateno C, Yoshizane Y, Saito N, Kataoka M, Utoh R, et al (2004) Near completely humanized liver in mice shows human-type metabolic responses to drugs. Am J Pathol 165: 901–912. 15331414 10.1016/S0002-9440(10)63352-4 PMC1618591

[20] Takeuchi T, Katsume A, Tanaka T, Abe A, Inoue K, et al (1999) Real-time detection system for quantification of hepatitis C virus genome. Gastroenterology 116: 636–642. 10029622 10.1016/s0016-5085(99)70185-x

[21] Tanaka T, Inoue K, Hayashi Y, Abe A, Tsukiyama-Kohara K, et al (2004) Virological significance of low-level hepatitis B virus infection in patients with hepatitis C virus associated liver disease. J Med Virol 72: 223–229. 14695663 10.1002/jmv.10566

[22] Kashiwakuma T, Hasegawa A, Kajita T, Takata A, Mori H, et al (1996) Detection of hepatitis C virus specific core protein in serum of patients by a sensitive fluorescence enzyme immunoassay (FEIA). J Immunol Methods 190: 79–89. 8601714 10.1016/0022-1759(95)00261-8

[23] Takaoka A, Wang Z, Choi MK, Yanai H, Negishi H, et al (2007) DAI (DLM-1/ZBP1) is a cytosolic DNA sensor and an activator of innate immune response. Nature 448: 501–505. 17618271 10.1038/nature06013

[24] Sakamoto H, Okamoto K, Aoki M, Kato H, Katsume A, et al (2005) Host sphingolipid biosynthesis as a target for hepatitis C virus therapy. Nat Chem Biol 1: 333–337. 16408072 10.1038/nchembio742

[25] Watanabe T, Sudoh M, Miyagishi M, Akashi H, Arai M, et al (2006) Intracellular-diced dsRNA has enhanced efficacy for silencing HCV RNA and overcomes variation in the viral genotype. Gene Ther 13: 883–892. 16496015 10.1038/sj.gt.3302734

[26] Minakuchi Y, Takeshita F, Kosaka N, Sasaki H, Yamamoto Y, et al (2004) Atelocollagen-mediated synthetic small interfering RNA delivery for effective gene silencing in vitro and in vivo. Nucleic Acids Res 32: e109. 15272050 10.1093/nar/gnh093 PMC506824

[27] Gidlund M, Orn A, Wigzell H, Senik A, Gresser I (1978) Enhanced NK cell activity in mice injected with interferon and interferon inducers. Nature 273: 759–761. 566386 10.1038/273759a0

[28] Djeu JY, Heinbaugh JA, Holden HT, Herberman RB (1979) Role of macrophages in the augementation of mouse natural killer cell activity by poly I:C and interferon. J Immunol 122: 182–188. 216746

[29] Kotenko SV, Gallagher G, Baurin VV, Lewis-Antes A, Shen M, et al (2003) IFN-lambdas mediate antiviral protection through a distinct class II cytokine receptor complex. Nat Immunol 4: 69–77. 12483210 10.1038/ni875

[30] Sheppard P, Kindsvogel W, Xu W, Henderson K, Schlutsmeyer S, et al (2003) IL-28, IL-29 and their class II cytokine receptor IL-28R. Nat Immunol 4: 63–68. 12469119 10.1038/ni873

[31] Prokunina-Olsson L, Muchmore B, Tang W, Pfeiffer RM, Park H, et al (2013) A variant upstream of IFNL3 (IL28B) creating a new interferon gene IFNL4 is associated with impaired clearance of hepatitis C virus. Nat Genet 45: 164–171. 23291588 10.1038/ng.2521 PMC3793390

[32] Pott J, Mahlakoiv T, Mordstein M, Duerr CU, Michiels T, et al (2011) IFN-lambda determines the intestinal epithelial antiviral host defense. Proc Natl Acad Sci U S A 108: 7944–7949. 21518880 10.1073/pnas.1100552108 PMC3093475

[33] Iversen MB, Ank N, Melchjorsen J, Paludan SR (2010) Expression of type III interferon (IFN) in the vaginal mucosa is mediated primarily by dendritic cells and displays stronger dependence on NF-kappaB than type I IFNs. J Virol 84: 4579–4586. 20181703 10.1128/JVI.02591-09 PMC2863761

[34] Mordstein M, Neugebauer E, Ditt V, Jessen B, Rieger T, et al (2010) Lambda interferon renders epithelial cells of the respiratory and gastrointestinal tracts resistant to viral infections. J Virol 84: 5670–5677. 20335250 10.1128/JVI.00272-10 PMC2876583

[35] Mordstein M, Kochs G, Dumoutier L, Renauld JC, Paludan SR, et al (2008) Interferon-lambda contributes to innate immunity of mice against influenza A virus but not against hepatotropic viruses. PLoS Pathog 4: e1000151. 18787692 10.1371/journal.ppat.1000151 PMC2522277

[36] Kumar H, Kawai T, Kato H, Sato S, Takahashi K, et al (2006) Essential role of IPS-1 in innate immune responses against RNA viruses. J Exp Med 203: 1795–1803. 16785313 10.1084/jem.20060792 PMC2118350

[37] Li K, Chen Z, Kato N, Gale M Jr, Lemon SM (2005) Distinct poly(I-C) and virus-activated signaling pathways leading to interferon-beta production in hepatocytes. J Biol Chem 280: 16739–16747. 15737993 10.1074/jbc.M414139200

[38] Wang N, Liang Y, Devaraj S, Wang J, Lemon SM, et al (2009) Toll-like receptor 3 mediates establishment of an antiviral state against hepatitis C virus in hepatoma cells. J Virol 83: 9824–9834. 19625408 10.1128/JVI.01125-09 PMC2747996

[39] Li K, Foy E, Ferreon JC, Nakamura M, Ferreon AC, et al (2005) Immune evasion by hepatitis C virus NS3/4A protease-mediated cleavage of the Toll-like receptor 3 adaptor protein TRIF. Proc Natl Acad Sci U S A 102: 2992–2997. 15710891 10.1073/pnas.0408824102 PMC548795

[40] Meylan E, Curran J, Hofmann K, Moradpour D, Binder M, et al (2005) Cardif is an adaptor protein in the RIG-I antiviral pathway and is targeted by hepatitis C virus. Nature 437: 1167–1172. 16177806 10.1038/nature04193

[41] Bellecave P, Sarasin-Filipowicz M, Donze O, Kennel A, Gouttenoire J, et al (2010) Cleavage of mitochondrial antiviral signaling protein in the liver of patients with chronic hepatitis C correlates with a reduced activation of the endogenous interferon system. Hepatology 51: 1127–1136. 20044805 10.1002/hep.23426

