# Supplementary material for: Targeted Induction of Interferon-λ in Humanized Chimeric Mouse Liver Abrogates Hepatotropic Virus Infection
Source: PLoS One. 2013 Mar 28;8(3):e59611. doi: 10.1371/journal.pone.0059611 (PMC3610702; doi:10.1371/journal.pone.0059611)
Supplement: Text S1 — Supporting materials, methods, and references. Methods for “Biodistribution of nucleic acids complexed with LIC”, “Measurement of human serum albumin”, “Measurement of alanine aminotransferase”, “Enzyme-linked immunosorbent assay”, “Luciferase and WST-8 assays of HCV replicon cells”, “Depletion of NK and natural killer T cells”, “Activation of NKT cells”, “Neutralizing activity of the anti-human IFN-λ antibody”, and “SNP genotyping of IFNL3 (IL28B)”, along with supporting references, are provided in the Supporting Materials section. (DOC) [file pone.0059611.s012.doc]

**Supporting materials and methods**

***Biodistribution of nucleic acids complexed with LIC.***

A complex comprised of ^3^H-labeled short dsRNA and LIC was intravenously administered to normal ddY mice at a dose of 10 mg/kg. After 5, 15 or 60 min, the animals were sacrificed by whole blood collection under isoflurane (Abbott, Chicago, IL, USA) anesthesia and their lungs, heart, liver, spleen and kidneys were removed and minced in Solvable (PerkinElmer, Waltham, MA, USA), an alkaline aqueous-based tissue solubilizer. After overnight incubation at 40°C, samples were dissolved in Hionic-Fluor (PerkinElmer) and radioactivity was measured in a Tri-Carb 2500TR liquid scintillation analyzer (PerkinElmer).

***Measurement of human serum albumin.***

Human serum albumin in the blood of the chimeric mice was measured using the Alb-II kit (Eiken Chemical, Tokyo, Japan) according to the manufacturer’s instructions.

***Measurement of alanine aminotransferase.***

Serum alanine aminotransferase (ALT) levels were measured using a Transaminase-CII Test A kit (Wako Pure Chemical Industries, Osaka, Japan).

***Enzyme-linked immunosorbent assay***

IFN-α and IFN-β concentrations in chimeric mouse serum were measured using a human IFN-β enzyme-linked immunosorbent assay (ELISA) Kit (TFB, Tokyo, Japan), human IFN-α ELISA Kit (PBL InterferonSource, Piscataway, NJ, USA), mouse IFN-β ELISA Kit (PBL InterferonSource) or mouse IFN-α ELISA Kit (PBL InterferonSource), as appropriate. IFN-λ1 (IL-29) and IFN-λ2 (IL-28A) concentrations in chimeric mice serum and in the culture medium were measured using an IL-29 and IL-28A ELISA Kit (R&D Systems). In all cases, we followed the manufacturers’ instructions.

***Luciferase and WST-8 assays of HCV replicon cells***

The HCV replicon cells were seeded in 96-well plates at a density of 4 × 10^3^ cells/well. The next day, recombinant human or mouse IFN-β (PBL InterferonSource) or human IFN-λ was added to the culture medium. After 72 h incubation, luciferase assays were performed by using a Steady-Glo luciferase assay kit (Promega, Madison, WI, USA). Cell viability was measured using 2-(2-methoxy-4-nitrophenyl)-3-(4-nitrophenyl)-5-(2,4-disulfophenyl)-*2H*-tetrazolium (WST-8; Cell Counting Kit-8, Dojindo, Kumamoto, Japan) according to the manufacturer’s instructions.

***Depletion of NK and natural killer T cells.***

We depleted NK and natural killer T (NKT) cells in chimeric mice by using an anti-IL2Rβ monoclonal antibody, TM-β1 [42], which recognizes the mouse IL-2 receptor β-chain. Four days before the first LIC-pIC administration, 1 mg of TM-β1 in saline (200 μl) was injected intraperitoneally.

***Activation of NKT cells.***

A specific activator of NKT cells, α-galactosylceramide, was intravenously administered to the chimeric mice at a dose of 1 μg/kg weekly. The dosing volume was 10 μl/g of body weight.

***Neutralizing activity of the anti-human IFN-λ antibody.***

FLR3-1 HCV replicon cells that express a luciferase reporter plasmid were seeded in 96-well plates at a density of 4 × 10^3^ cells/well. The next day, anti-human IFN-λ1 (R&D Systems) or anti-human IFN-λ2 (R&D Systems) antibodies were added to the culture medium at a concentration of 1 μg/ml or 10 μg/ml. After 6 h incubation, recombinant human IFN-λ1, IFN-λ2 or IFN-λ3 was added to the culture medium at a concentration of 100 ng/ml. After 48 h incubation, luciferase assays were performed.

***SNP genotyping of IFNL3 (IL28B)***

The genomic DNA sequences of donor hepatocytes of humanized liver mice were determined using a 3730 DNA analyzer (Applied Biosystems) as previously described [10].

**Supporting references**

**[42] Tanaka T, Kitamura F, Nagasaka Y, Kuida K, Suwa H, Miyasaka M (1993) Selective long-term elimination of natural killer cells in vivo by an anti-interleukin 2 receptor beta chain monoclonal antibody in mice. J Exp Med 178: 1103**–**1107.**
